# Disturbed lipid profile in common variable immunodeficiency – a pathogenic loop of inflammation and metabolic disturbances

**DOI:** 10.3389/fimmu.2023.1199727

**Published:** 2023-07-20

**Authors:** Silje F. Jorgensen, Magnhild E. Macpherson, Tonje Skarpengland, Rolf K. Berge, Børre Fevang, Bente Halvorsen, Pål Aukrust

**Affiliations:** ^1^ Research Institute of Internal Medicine, Oslo University Hospital Rikshospitalet, Oslo, Norway; ^2^ Section of Clinical Immunology and Infectious Diseases, Oslo University Hospital Rikshospitalet, Oslo, Norway; ^3^ Department of Clinical Science, University of Bergen, Bergen, Norway; ^4^ Department of Heart Disease, Haukeland University Hospital, Bergen, Norway; ^5^ Institute of Clinical Medicine, University of Oslo, Oslo, Norway

**Keywords:** CVID - common variable immunodeficiency, antibody deficiencies, fatty acids, metabolism, inflammation, HDL - cholesterol, triglycerid

## Abstract

The relationship between metabolic and inflammatory pathways play a pathogenic role in various cardiometabolic disorders and is potentially also involved in the pathogenesis of other disorders such as cancer, autoimmunity and infectious diseases. Common variable immunodeficiency (CVID) is the most common primary immunodeficiency in adults, characterized by increased frequency of airway infections with capsulated bacteria. In addition, a large proportion of CVID patients have autoimmune and inflammatory complications associated with systemic inflammation. We summarize the evidence that support a role of a bidirectional pathogenic interaction between inflammation and metabolic disturbances in CVID. This include low levels and function of high-density lipoprotein (HDL), high levels of triglycerides (TG) and its major lipoprotein very low-density lipoprotein (VLDL), and an unfavorable fatty acid (FA) profile. The dysregulation of TG, VLDL and FA were linked to disturbed gut microbiota profile, and TG and VLDL levels were strongly associated with lipopolysaccharides (LPS), a marker of gut leakage in blood. Of note, the disturbed lipid profile in CVID did not include total cholesterol levels or high low-density lipoprotein levels. Furthermore, increased VLDL and TG levels in blood were not associated with diet, high body mass index and liver steatosis, suggesting a different phenotype than in patients with traditional cardiovascular risk such as metabolic syndrome. We hypothesize that these metabolic disturbances are linked to inflammation in a bidirectional manner with disturbed gut microbiota as a potential contributing factor.

## Introduction

Metabolic disturbances are thoroughly studied in classical metabolic disorders such as diabetes mellitus and various cardiometabolic diseases, uncovering strong links to inflammation which promotes disease progression (1–3). The relationship between metabolic and inflammatory pathways has also been studied in other disorders such as cancer, autoimmunity and infectious diseases, where it could potentially contribute to disease progression (4–8).

Common variable immunodeficiency (CVID) is the most common primary immunodeficiency in adults, affecting 1: 25 000 to 1: 50 000 Caucasians (9). CVID patients have a B-cell dysfunction leading to hypogammaglobulinemia and typically increased frequency of infections with capsulated bacteria in airways (10). In addition, 70-80% of CVID patients have non-infectious complication e.g., autoimmunity, lymphadenopathy, splenomegaly, granulomas, cytopenia and enteropathy, associated with increased mortality and morbidity (11). These autoimmune and inflammatory complications are linked to low-grade systemic inflammation and immune activation with abnormal T cell and monocyte/macrophage function (12–14). Moreover, during the recent years, some studies have shown that CVID patients have an altered composition of gut microbiota that could be related to systemic inflammation (15–17). So far, however, data on metabolic disturbances in CVID patients and their potential relationship to CVID phenotypes, systemic inflammation and gut microbiota composition are scarce. In this review article, we will present existing data on metabolic abnormalities in CVID and how these disturbances could potentially be an important bridge, linking gut microbiota to systemic inflammation and autoimmunity in these patients.

## Brief overview of lipid metabolism

**Figure 1:**
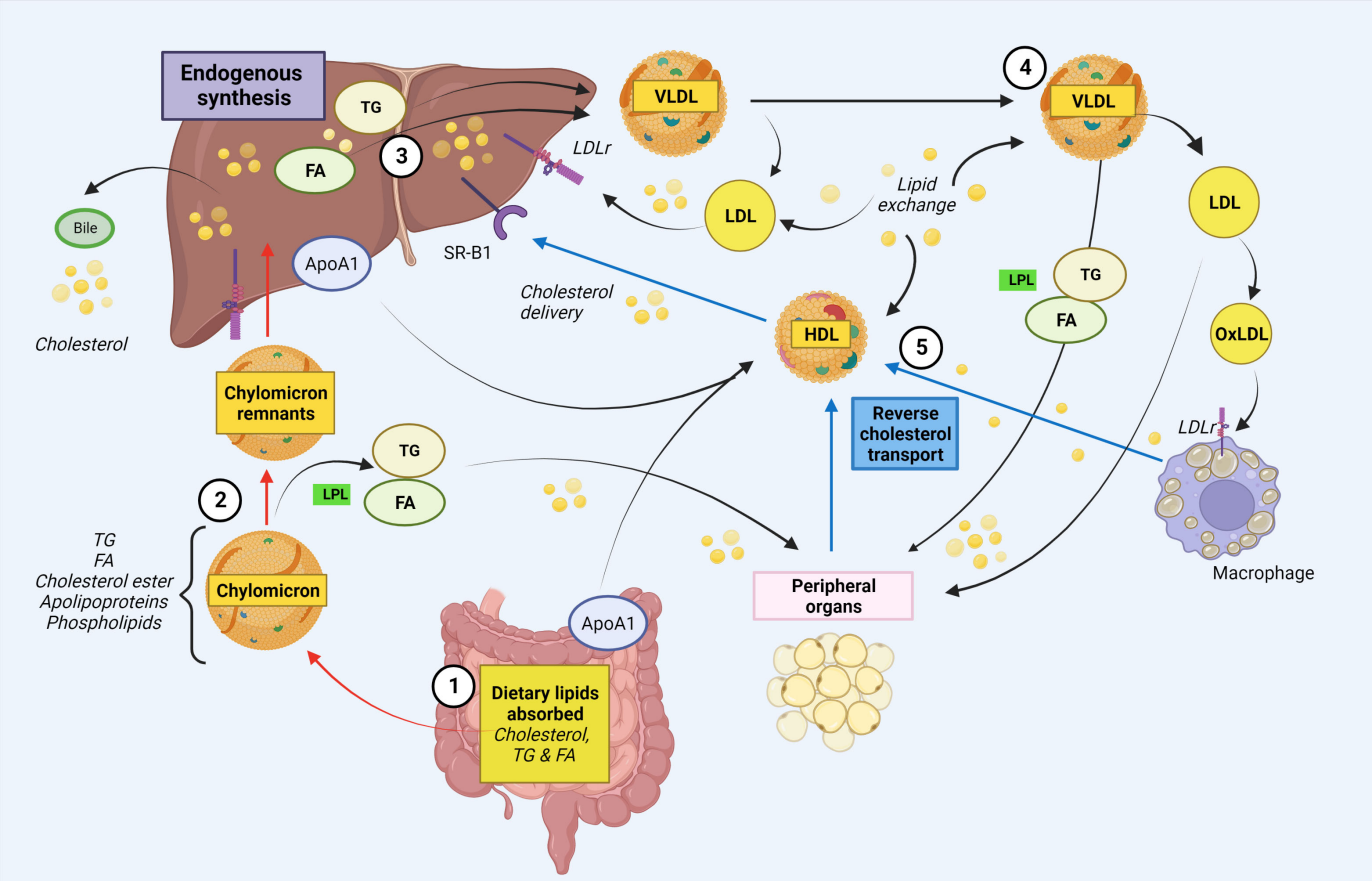
Simplified schematic illustration of cholesterol and fatty acid metabolism. **1.** Intestinal absorption of dietary lipids. Cholesteryl esters, free fatty acids (FA) and triglycerides (TG) from the diet are absorbed in the intestinal lumen. In the enterocytes, chylomicrons are formed, which enters the circulation via the lymphatic system. In addition, ApoA1, the major protein component of HDL, is endogenous synthetized in enterocytes (and liver). **2.** Chylomicrons deliver lipids to peripheral organs. Within the Chylomicrons, TGs are hydrolyzed by lipoprotein lipase (LPL) in peripheral organs, releasing FAs for storage. **3.** The assembly of VLDL in the liver. The chylomicron remnants containing cholesterol are taken up by the liver. The liver delivers both endogenously synthesized and exogenously acquired FAs. FAs are re-esterified to form triglyceride and together with cholesterol form VLDL. VLDL enters the bloodstream as triglyceride rich lipoprotein VLDL. The liver also eliminate cholesterol via bile/intestine. **4.** VLDLs transport triglycerides to peripheral tissues. Via the blood stream, VLDL can transport TGs to peripheral cells e.g. adipose tissue and muscle, where it is hydrolyzed by LPL to FAs for energy production or storage. VLDL can also generate circulating LDL which can either return to the liver or it can be oxidized and taken up by macrophages in peripheral organs. Macrophages loaded with excess cholesterol become foam cells. **5.** Cholesterol is recycled or eliminated through reverse cholesterol transport. HDL cholesterol is transported from peripheral cells/tissues back to the liver and intestine, where cholesterol is recycled or eliminated. HDL cholesterol can reach the liver either directly through binding to SR-B1, or indirectly after a lipid exchange with VLDL/LDL, which are cleared by hepatic LDL receptor. Red arrows indicate exogenous pathways, black arrows indicate endogenous pathways and blue arrows indicate reverse cholesterol transport. ApoA-I, apolipoprotein A-I; FA, fatty acid; HDL, high-density lipoprotein, LDLr, low-density lipoprotein receptor; LPL, lipoprotein lipase; oxLDL, oxidized low-density lipoprotein; SR, scavenger receptor; TG, triglycerides; VLDL, very low-density lipoprotein. Created with BioRender.com.

Cholesterol and its esterified form, cholesteryl esters, are essential to form and maintain cell membranes, and is also crucial for modulation of transmembrane signaling pathways, as well as for the synthesis of hormones, fat-soluble vitamins and production of bile acids. Although it is biosynthesized in all mammalian cells, humans mainly obtain cholesterol from endogenous production in the liver and intestines, as well as from the diet through intestinal absorption (18–20). Cholesterol absorption starts with micellar solubilization of nutrient cholesterol in the intestinal lumen, before transportation to the enterocyte brush border membrane where it interacts with the enterocyte and gains entry (20, 21). Once cholesterol has been absorbed, it undergoes intracellular esterification into cholesteryl esters. Within the enterocyte, diet-derived fatty acids (FAs) are re-esterified to form triglycerides (TGs), and packaged with apolipoproteins, cholesteryl esters, free cholesterol and phospholipids to form chylomicron particles for secretion at the basolateral surface of enterocytes (20, 22). Chylomicrons then enter the circulation *via* the lymphatic system, and transport its lipids to skeletal, muscle, cardiac and adipose tissues where their TG components are hydrolyzed by capillary lipoprotein lipase (LPL) to release free FAs for storage or oxidation (23) (**Figure 1**). Interesting, a recent study suggest that intestinal synthesized HDL particles containing ApoA-I, are generated and released in the portal vein (24). As the FA-depleted, cholesterol-rich chylomicron remnants reach the liver, they are cleared from the circulation by the hepatic low-density lipoprotein (LDL) receptor-related protein. From the liver, recycled and endogenously produced cholesterol can enter the metabolic pathway as very low-density lipoprotein (VLDL), of which the TG core is hydrolyzed to produce intermediate-density lipoprotein (IDL) (25). The IDL particles are taken up by the liver or further hydrolyzed to produce LDL. Oxidized LDL can enter macrophages *via* scavenger receptors (SR) CD36 and SR-A, that is not downregulated by intracellular cholesterol levels, and join the intracellular cholesterol pool in an unregulated way. These cells also contain LDL receptors that is regulated by intracellular cholesterol levels. Macrophages loaded with excess cholesterol become foam cells, a hallmark in the pathogenesis of disorders characterized by an interaction between lipids and inflammation such as atherosclerosis (26, 27). HDL cholesterol is transported from peripheral cells/tissues back to the liver and intestine, where cholesterol is recycled or eliminated through reverse cholesterol transport (28). HDL cholesterol can reach the liver either directly through binding to SR-B1, or indirectly after a lipid exchange with VLDL/LDL, which are cleared by hepatic LDL receptor (28). A simplified illustration of lipid metabolism is shown in **Figure 1**.

Whereas normal lipid metabolism is essential for normal cellular function and homeostasis, abnormal regulation of these pathways may through a bidirectional interaction with immune cells have major implication for several disorders, not only atherosclerosis and related metabolic disorders, but also various disorders characterized by systemic inflammation and autoimmunity. A significant proportion of CVID patients develop inflammatory and autoimmune complications, and herein, we demonstrate support for our hypothesis that an unfavorable/dysregulated metabolic profile is linked to the inflammatory phenotype in CVID. The central role of the intestine in lipid metabolism, suggest that gut microbial composition may also influence lipid metabolism. Therefore, potential mechanisms linking gut microbiota to lipid metabolism and inflammation in CVID (29), will also be discussed in this review.

## Low levels of high-density lipoprotein in CVID: potential contribution to systemic inflammation

It is well known that HDL has anti-atherogenic properties that are primarily thought to be attributed to its key role in reverse cholesterol transport (30, 31). However, whereas most lipoproteins are thought to mediate inflammatory effects, several lines of evidence suggest that HDL has anti-inflammatory properties that could also contribute to its anti-atherogenic potential (32, 33). Thus, HDL have been shown to mediate inhibitory effects on several pathways relevant for immune activation and inflammation, at least partly through inhibitory effects on innate immunity, interacting both with toll-like receptors (TLR), such as TLR4 and TLR2, and NLRP inflammasomes, all central regulators of the innate immune responses in monocytes/macrophages (33–37).

Vieira et al. was the first to report deceased circulating levels of HDL cholesterol and Apolipoprotein A-I (ApoA-I), the major protein component in HDL, in a combined cohort of patients with CVID (n=18) and X-linked agammaglobulinemia (XLA, n=6), compared to healthy controls (38). More recently, Andrade et al. reported decreased levels of ApoA-I associated with increased oxidative stress in 32 CVID patients (39). MacPherson et al. went on to show decreased levels of HDL levels in a large cohort of 102 CVID patients (40). In a sub-analysis of 40 patients they also found decreased ApoA-1 levels compared to controls. In addition, they analyzed HDL subclasses, defined by density or size reflecting differences in relative content of proteins and lipids, and found decreased levels of extra-large, large and medium sized HDL, but not of the small sized HDL, compared to healthy controls (40). The small HDL subclass has been suggested to be of particular importance for reverse cholesterol transport (41), but how the actual HDL subclass pattern in CVID affects its function in these patients is at present unclear. Moreover, in the study by MacPherson et al., HDL levels were inversely correlated with tumor necrosis factor (TNF) and C-reactive protein (CRP), both markers of systemic inflammation (40).

**Figure 2:**
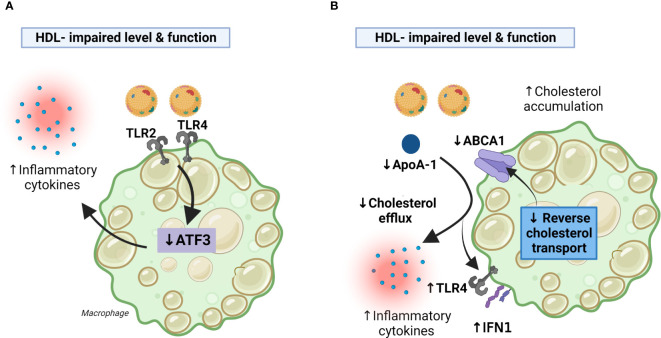
Decreased HDL levels and function in CVID may facilitate inflammatory responses in macrophages. Panel **(A)** HDL exert anti-inflammatory effects in macrophages by the induction of ATF3. CVID patients with systemic inflammation autoimmunity and inflammation have impaired HDL levels and function along with decreased AFT3 expression resulting in enhanced TLR2/4 signaling and increased production of inflammatory cytokines. Panel **(B)** CVID patients with autoimmunity and systemic inflammation had impaired cholesterol efflux capacity along with decreased expression of ABCA1 (PBMC) an ApoA-1 (serum), suggesting impaired RCT. This may result in (i) less inhibition of TLR4 and type I IFN signaling caused by attenuated RCT, (ii) less inhibition of TLR4 mediated release of inflammatory cytokines caused by down-regulation of ApoA-I and ABCA1. ABCA1, ATP-binding cassette transporter A1; ApoA-I, apolipoprotein A1; PBMC, peripheral blood mononuclear cells; RCT, reverse cholesterol transport; TLR, toll-like receptor. Created with BioRender.com.

While it is known that HDL can sequester lipopolysaccharide (LPS), and thereby prevent LPS-induced cellular activation through TLR4 (42), this mechanism alone does not explain why HDL can block a broad spectrum of TLR-mediated inflammatory responses in various cells such as macrophages. De Nardo E et al. showed that the induction of ATF3, a key transcriptional repressor of innate immune response genes, is the main mechanism by which HDL mediates its anti-inflammatory activities in macrophages (43). ATF3 can be induced by TLR activation as part of an important negative feedback loop to limit TLR-driven inflammatory responses in macrophages, and by inducing ATF3 (44), HDL utilizes an ancient regulatory feedback system to broadly and directly modify the inflammatory response of macrophages towards TLR stimulation (43). It seems that AFT3 is a more general regulator of inflammation and cell metabolisms with relevance to a wide range of diseases ranging from metabolic disorders to cancer (45). Interestingly, in the study by MacPherson et al. it was reported decreased expression of ATF3 in peripheral blood mononuclear cells (PBMC) from CVID patients, and PBMC from CVID patients had impaired anti-inflammatory effects of HDL in these cells, suggesting that attenuated HDL-AFT3 interaction could contribute to systemic inflammation in CVID (40) (**Figure 2A**).

In the study by Macpherson et al., it was shown that monocyte-derived macrophages from CVID patients had impaired cholesterol efflux capacity along with decreased expression ATP-binding cassette transporter A1 (ABCA1), a cell membrane protein that exports excess cholesterol from cells to ApoA-I, in their PBMC, suggesting impaired reverse cholesterol transport (40). Reverse cholesterol transport is an important part of the athero-protective properties of HDL (31), and it has also been shown that this process mediates anti-inflammatory effects. Thus, by counteracting cholesterol accumulation in macrophages, reverse cholesterol transport seems to inhibit TLR4 and type I interferon (IFN) signaling (37). Moreover, in addition to be of importance for cholesterol efflux mechanism, the interaction of apoA-I with ABCA1-expressing macrophages seems to suppress the ability of LPS to induce the inflammatory cytokines like interleukin (IL)-1β, IL-6, and TNF involving JAK2/STAT3 activation (46). Thus, it is plausible that the impaired cholesterol efflux mechanism, that involves impaired function and levels of HDL, decreased levels of ApoA-I (serum) and ABCA1 (macrophages) in CVID patients could contribute to the inflammatory phenotype in subgroups of these patients (**Figure 2B**). However, it has also been suggested that HDL could exert inflammatory effects in macrophages *via* passive cholesterol depletion and enhancement of TLR-induced signaling through a protein kinase C/NFkB/STAT1-IRF1 axis (38). These discrepancies could reflect differences in the experimental setup (e.g*., in vivo* versus *in vitro* experiments), but notably, this phenomenon could also reflect structural changes in the HDL particles during certain disease categories that include also chronic inflammatory disorders and the interaction between HDL and macrophages is still not fully elucidated (47, 48).

**Figure 3:**
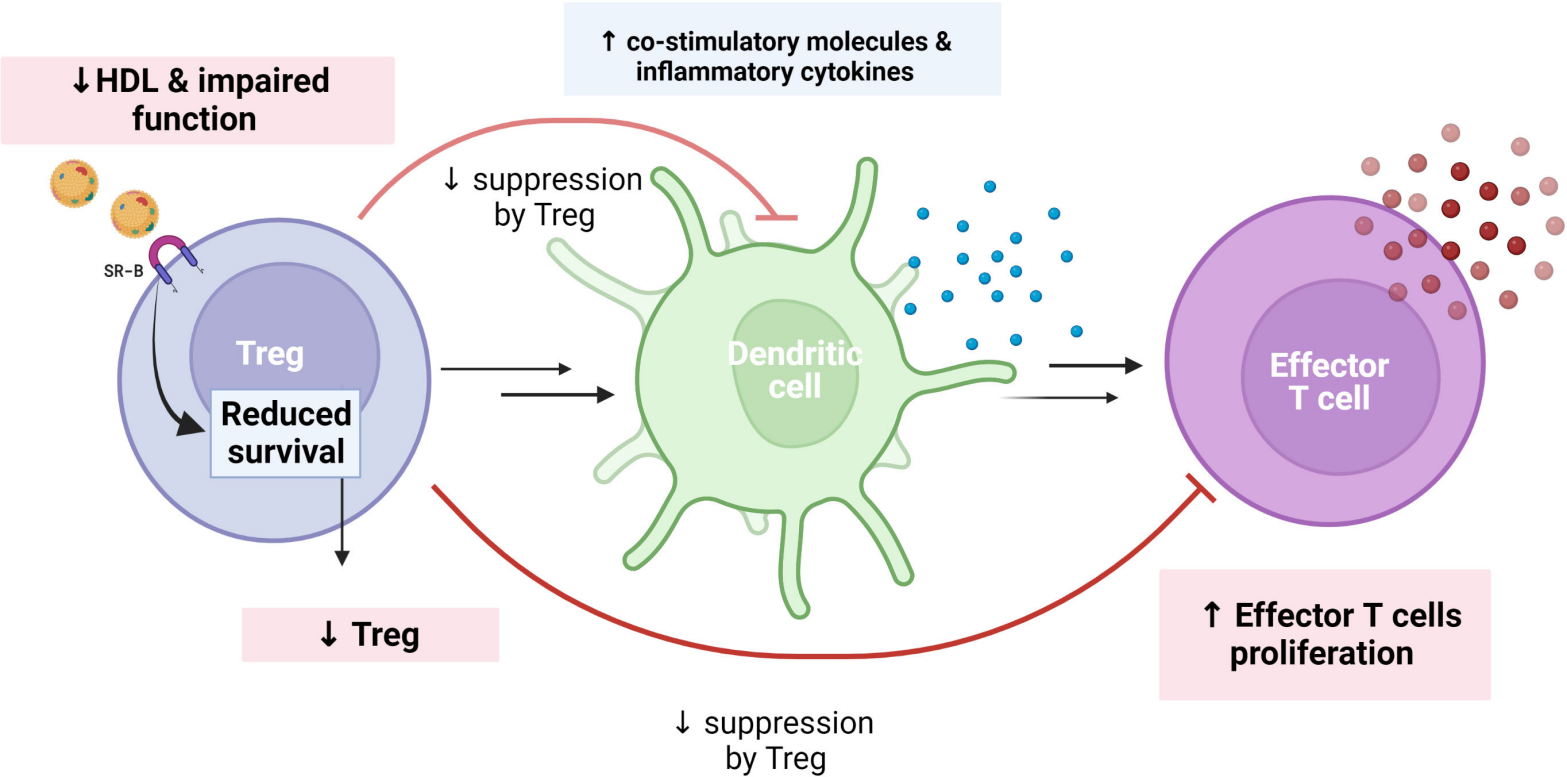
Theoretical figure showing how reduced HDL levels/function may explain reduce number/function of regulatory T cells (Treg) in CVID. In contrast to LDL, HDL is taken up in Tregs through the scavenger receptor SR-BI/BII, increasing the survival of this important T cell subset. Treg may attenuate dendritic cell response as well as proliferation of effector T cells to ensure a balanced immune response. This regulatory function of Tregs may be weakened in a situation with impaired HDL levels and function, as in CVID. We hypothesized that reduced HDL levels and function are associated with reduced Treg in CVID, which in turn decrease suppression of co-stimulatory molecules and increase effector T cell proliferation as well as inflammatory cytokines. Created with BioRender.com.

HDL and its main protein component ApoA-I can also exert anti-inflammatory effects by modulating T cell activation (49). Lipid rafts are regions of the plasma membrane which contain free cholesterol, sphingomyelin, and gangliosides that influence the functional capacity of both T and B cells (50). Enrichment of these lipid metabolites within cell membranes will enhance the cells autoimmune and inflammatory potential, and notably, HDL can attenuate these potential harmful properties through its enhancing effects on reverse cholesterol transport mechanisms (51). Of particular interest, regulatory T cells (T_reg_) are of major importance to ensure a balanced T cell response, interacting among others with dendritic cells (professional antigen-presenting cell type), to ensure a balanced immune response and notably (52–54), CVID patients have been shown to have a decreased proportion of this T cell subtype (55). In contrast to LDL, HDL seems to be taken up in T_reg_ through scavenger receptor SR-BI/BII, and this appear to increase the survival of this important T cell subset (51, 56, 57). Thus, one could hypothesize that the decreased levels and impaired function of HDL in CVID could, at least partly, contribute to the decreased number of T_reg_ and potentially also impaired function resulting in activated dendritic cells as well as enhanced production of effector T cells (**Figure 3**). In the largest HDL study on CVID, step-wise regression analysis identified soluble CD25, a reliable marker of T cell activation, as well as sex as the strongest predictor of HDL levels in CVID (40), further supporting a link between T cell pathology and low HDL levels in these patients.

Data on the influence of HDL on B cell function are scarce. However, based on the ability of HDL to influence lipid rafts composition it is conceivable that this also will affect B cell function (58), and these issues will have to be clarified through further studies on the CVID population.

## High levels of triglycerides and very-low density lipoprotein in CVID: link to systemic inflammation and an autoimmune phenotype

In contrast to HDL, which has both athero-protective and anti-inflammatory properties, TG and its major lipoprotein carrier, VLDL, are regarded as pro-atherogenic and related to inflammation (59–61). Although Vieira et al. found an inverse correlation between HDL and TG in CVID and XLA patients (n=18 and n=6, respectively), they found no differences in TG levels between these patients and healthy controls (38). More recently, however, Macpherson et al. reported significantly raised levels of TG in 95 CVID patients as compared with healthy controls (62). In a sub-study of 40 CVID patients, VLDL levels including all six sizes of this lipoprotein (extra-extra-large to extra-small), were also found to be increased as compared with healthy controls (62). It is well known that TG and VLDL are increased during acute infections (63, 64), but in the CVID study patients with ongoing acute infections were excluded (62). Moreover, TG and VLDL levels were not related to the occurrence of bronchiectasis, a condition that is characterized by low-grade and recurrent exacerbations of bacterial infections in the lower airways. In contrast, TG levels were significantly increased in the subgroup of CVID patients with autoimmunity and sterile inflammatory complications as compared to CVID patients without these complications (i.e., “infection only”) (62). TG levels in CVID patients were also correlated with increased levels of inflammatory markers (i.e., CRP, IL-6 and IL-12). Interestingly, there was a strong positive correlation between plasma levels of TG and LPS, linking disturbed VLDL/TG metabolism to gut leakage mechanisms in these patients (62). Thus, rather than being related to ongoing bacterial infections, it seems that raised levels of TG and VLDL in CVID is related to autoimmunity and non-infectious inflammation, which could also involve gut leakage mechanisms.

**Figure 4:**
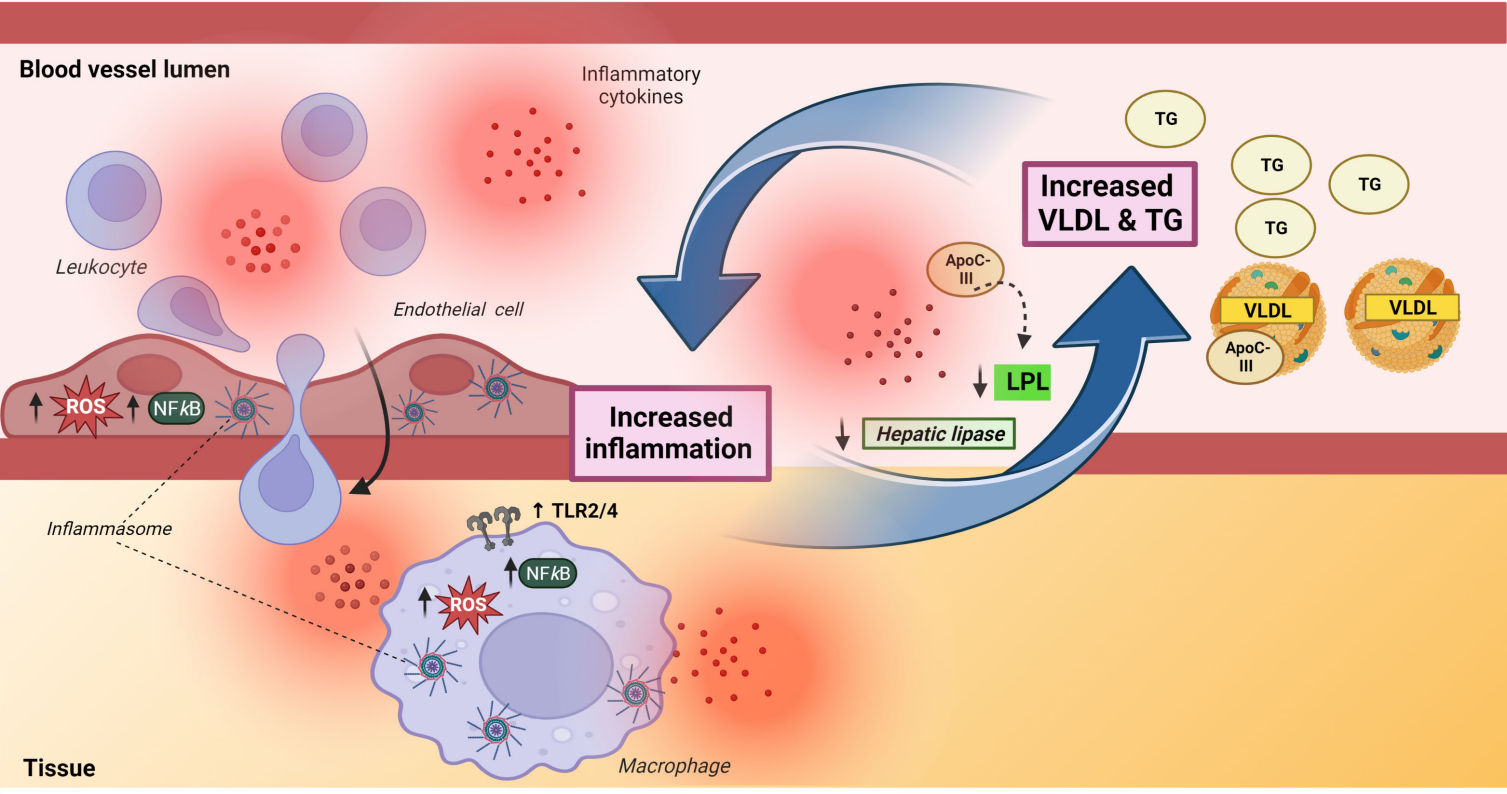
A bidirectional interaction between inflammation and triglycerides and very low-density lipoprotein. The left part of the Figure illustrates the complex effect of TG/VLDL on inflammation, inducing upregulation of TLR2/TLR4 in macrophages as well as promoting enhanced production of reactive oxygen species, activation of NFkB signaling pathways and activation of NLRP inflammasomes in both endothelial (NLRP1) and macrophages (NLRP3). The latter involves ApoC-III related mechanisms. The activation of endothelial cells facilitates leukocyte migration into inflamed tissue. The right part of the Figure illustrates that inflammation increases TG/VLDL levels by downregulating levels and attenuating the lipolytic activity of the enzymes, hepatic and lipoprotein lipase. Apoliprotein C-III, a major component of VLDL, could also attenuate the lipolytic activity of lipoprotein lipase. ApoC-III, apolipoprotein C-III; HL, hepatic lipase; LPL, lipoprotein lipase; ROS, reactive oxygen species, TG, triglycerides; VLDL, very low-density lipoprotein. Created with BioRender.com.

It is shown that inflammation could attenuate the effect and/or synthesis of enzymes that degrade TG such as lipoprotein lipase (LPL) and hepatic lipase (64, 65), and LPL has also been shown to enhance the levels of inflammatory cytokines like TNF (66). Moreover, the VLDL-associated lipoprotein ApoC-III has emerged as being particularly important as a regulator of TG transport involving mechanisms like impairment of LPL-mediated lipolysis, promotion of hepatic VLDL secretion, and suppression of TG clearance through the LDL receptor (67), and notably, ApoC-III may in itself promote inflammation (68). This displays the complexity of interactions between TG/VLDL metabolism and inflammation (**Figure 4**). Although it is tempting to hypothesize that persistent inflammation may downregulate LPL and hepatic lipase, whilst increasing ApoC-III in CVID, there is currently no data on these important regulators of TG and TG-rich lipoproteins (i.e., VLDL) in CVID.

Whereas inflammation may promote hypertriglyceridemia and increased levels of VLDL, these lipid metabolites may themselves also promote inflammation, potentially representing a pathogenic loop in inflammatory disorders like CVID with autoimmune and inflammatory complications (61, 69), **Figure 4**. Thus, VLDL has been reported to induce inflammatory responses such as increased release of IL-1β and TNF in monocytes/macrophages through various mechanisms like activation of AP-1, ERK and NFκB pathways (70–72). Of particular interest, Zewinger and colleagues showed that ApoC-III, a major constituent of VLDL and a modulator of TG metabolism, activated the NLRP3 inflammasome in human monocytes in an alternative manner through caspase-8 and dimerization of TLR2 and TLR4 (73). This alternative inflammasome activation in human monocytes is mediated by the TLR adapter protein SCIMP, triggering Lyn/Syk-dependent calcium entry and the production of reactive oxygen species (ROS), leading to activation of caspase-8. More recently it has been reported that this effect of ApoC-III is not dependent of its binding of VLDL (74). However, VLDL is still a source of ApoC-III, but the exact molecular mechanisms for the ApoC-III-mediated activation of NLRP3 still need to be fully elucidated (75). TGs and in particular TG-rich lipoproteins (i.e., VLDL) can also promote inflammatory responses in endothelial cells, including induction of ROS and activation of various transcriptional factors like NF*k*B (59). VLDL has also been shown to promote expression of NLRP1 inflammasomes in human arterial endothelial cells (76 *)*, and in relevance to endothelial cell activation, it has been reported that fibrin interacts with VLDL and its receptor to promote leukocyte transmigration through inhibition of the src kinase Fyn (77). Thus, TG and in particular VLDL have several inflammatory properties with relevance for CVID such as monocyte activation, induction of inflammatory cytokines, enhanced ROS production and leukocyte/endothelial cell interaction (**Figure 4**), all features that have been demonstrated in subgroups of CVID patients (78–81).

## Dysregulated fatty acid composition in CVID: potential role in inflammation?

FAs constitute a major component of TGs cholesteryl esters as well as phospholipids, serving among others as sources of energy and structural building blocks of cell membranes. Also, FAs and their derivatives take part in the regulation of several intracellular processes such as gene transcription, post-translational modification of proteins as well as direct modulation of enzyme activities as coactivators, thereby possessing a great potential to influence human health and disease (82). According to their degree of unsaturation, FAs have been classified into saturated FAs (SFAs), monounsaturated FAs and polyunsaturated FAs (PUFAs). In terms of inflammation, the most significant FA functions are attributed to PUFAs. These compounds contain more than one double bond in their carbon backbone and can be further categorized in various groups according to their chemical structure. The common classification of PUFAs as either *n (omega)-3* PUFA *or n-6* PUFA refers to the position of the first double bond relative to the methyl end of the carbon chain backbone (83).

Eicosanoids are a family of *n-6* and *n-3* PUFA metabolites synthesized by enzymatic oxygenation pathways, and includes prostaglandins, thromboxanes and leukotrienes. These classical eicosanoids, which can be biosynthesized in various cell types upon inflammatory signals, are well-documented regulators of pathological as well as physiological inflammation (84, 85). Of these, some of the best studied groups are eicosapentaenoic acid (EPA; 20:5 *n-3*), docosahexaenoate (DHA; 22:6 *n-3*), dihomo-γ-linolenic acid (DGLA; 20:3 *n-6*), and arachidonic acid (AA; 20:4 *n-6*). PUFA metabolites are involved in a variety of cellular functions involving modulation of several inflammatory pathways (85, 86). For instance, prostaglandins and leukotrienes execute their biological effects by activating their respective cell surface G protein-coupled receptors (84). Also, FA could modulate inflammation through “specific” FA receptors such as peroxisome proliferator-activated receptors (PPAR)y (82, 87)and affect signaling through established inflammatory receptors like TLRs (88). FAs could also modulate intracellular receptors/sensors that control inflammatory cell signaling such as NLRP3 inflammasomes (89) and NFkB (90). Thus, whereas the *n-3* PUFAs DHA and EPA inhibit TLR4 signaling and NLRP3 activation (89), certain SFAs such as palmitate, the most abundant SFA in plasma, induces NLRP3 inflammasome and TLR4 activation (91, 92).

Data on FAs in CVID have been scarce or lacking. Recently, however, Skarpengland et al. reported an unfavorable FA profile in plasma with reduced proportions of EPA and DHA, as well as the *n-6* PUFA Linoleic acid (LA) (93). Although still debated, several studies have proposed potential beneficial effects of increased *n-3* PUFAs on autoimmunity, inflammation and related metabolic disorders (83, 94, 95), suggesting that the reduced proportion of EPA and DHA in CVID patients may represent a disadvantageous FA profile that may affect inflammation and the metabolic pathways in these patients. In contrast, the role of *n-6* PUFA in health and disease appear less clear (96). Thus, whereas LA that was reduced in CVID (93) has been thought to promote inflammation, some studies have suggested beneficial and anti-inflammatory effects of this *n-6* PUFA (97, 98). Also, de Pablo et al. showed that erythrocyte levels of LA were inversely associated with the risk of rheumatoid arthritis, whereas no associations were observed for other *n-6* or *n-*3 PUFAs (99). At present, however, the clinical and immunological consequences of reduced LA (and EPA and DHA) in CVID are not clear. Thus, in contrast to the CVID study on HDL and TG (40, 62), Skarpengland et al. found no association between the potential unfavorable FA profile and any clinical subtypes including those with autoimmunity or any systemic markers of inflammation (93). This could potentially, at least partly, reflect that a lower number of patients were included in the FA study (n=39) as compared with the other (HDL and TG) (n=95-102). However, the authors reported that IgG levels correlated with plasma *n-3* PUFA proportion (93) and notably, it has been reported that *n-3* PUFA can boost B cell activation and antibody production potentially involving enhanced levels of Th2 cytokines (100). Although there was no difference in the proportion of AA between CVID patients and controls (93), this n*-6* PUFA contributes to the formation of both leukotrienes and prostaglandins (101, 102). Interestingly, we have previously showed increased cAMP/protein kinase A type 1 activation in T cells from CVID contributing to their impaired T cell function, and this pathway interacts with AA and its derivate (103, 104).

More recently, eicosanoid-related studies have identified new classes of FA-derived bioactive metabolites with potentially anti-inflammatory and pro-resolving effects. These pro-resolving lipid mediators, including resolvins, lipoxines, protecines and maresines, are all derived from specific *n-6* and *n-3* PUFA precursors, and could represent novel preventive and therapeutic targets (105, 106). However, at present, data on these specialized pro-resolving mediators in CVID are, to the best of our knowledge, lacking.

## Gut microbial dysbiosis: a potential player in the dysregulated lipid metabolism in CVID

Many inflammatory and metabolic diseases have been associated with gut microbial dysbiosis, but mechanistic studies are scarce in humans. For most diseases it is not known if the gut microbial dysbiosis is causing the disease *or* the result of the disease or a combination thereof. However, in mice-studies, the host-microbe interaction has been studied in more detail showing that microbes can regulate both immune and metabolic pathways (107–110). Due to the heterogeneity of CVID, no mouse model exists to study the host-microbe interaction in this disorder (111), and therefore all microbiota studies in CVID are in humans. A decade ago Shulzhenko et al. presented results from gut biopsies in three CVID patients, showing that the intestinal epithelium introduced its own protective mechanisms by upregulating INF-inducible immune response pathways and simultaneously repressing Gata4-related metabolic functions (112). More recently, a few studies have investigated the gut microbiota profile in CVID patients (15, 29, 113–118). Gut microbial imbalance in patients with CVID mainly includes changes in microbial diversity, a decrease in presumably beneficial bacteria, and an increase in pathobionts. Interestingly, in another study by Shulzhenko et al., CVID patients with enteropathy had reduced mucosal IgA expression in duodenal biopsies and more pathobionts bacteria such as *A. baumannii (* 113). The largest CVID study on gut microbiota to date reported a CVID-specific dysbiosis index that captured the dysbiosis seen in CVID (29). This index consisted of ten taxa: *Bacilli, Dorea, Roseburia*, *Gammaproteobacteria* (increased in CVID), and *Bifidobacterium, Odoribacteracea, Christensenellaceae, Blautia, Sutterella, Desulfovibrionacea* (reduced in CVID). Subgroup analysis showed that gut microbial dysbiosis was significantly more pronounced in CVID patients with inflammatory and autoimmune complications than in CVID patients without. Furthermore, there was a clear positive association between markers of systemic inflammation in blood, representing activation of monocyte/macrophage (soluble CD14) and T cells (soluble CD25), and the degree of dysbiosis in stool samples (29). However, the role of gut microbiota in the pathogenesis of CVID is still not clear.

The relationship between gut microbiota and metabolic disturbances have been widely examined in particular in relation to cardiovascular and related metabolic disorders (119–124). A study by Fu et al. showed that gut microbiota contributed to lipid variation, independently of age, gender and genetics of the host (121). Notably, the association between gut microbial dysbiosis and lipids was particularly strong for HDL and TG, but weaker for LDL and total cholesterol. Moreover, whereas several taxa seemed to have an unfavorable effect on the lipid profile, certain orders (*Bacteroidales* [phylum *Bacteroidetes]* and family *Clostridiaceae* [phylum *Firmicutes*]), known to be involved in bile acid metabolism, were suggested to have a potential favorable effect. Both taxa are also involved in short chain FA acid (SCFA) metabolism and these microbiota-derived FAs seem to be protective against atherosclerosis and related metabolic complications like metabolic syndrome and type 2 diabetes mellitus (121, 125).

Data on the association between altered gut microbiota and lipid disturbances in CVID are scarce. However, Skarpengland et al. showed that the gut microbial alpha diversity was positively associated with plasma *n-6* PUFAs, but not with *n-3* PUFA, suggesting that plasma FA composition in blood could be related to gut microbial diversity (93). Interestingly, the FA composition was not related to the diet (93). Furthermore, a 2-week intervention with the non-absorbable antibiotic rifaximin, significantly altered FA composition in blood (29, 93, 126). These findings further support an important concept; that alteration of the gut microbial composition could affect FA levels in blood, at least in CVID.

In a recent study, Macpherson et al. reported a relationship between the CVID specific dysbiosis index in stools samples and TG levels in blood. Also, the major TG related lipoprotein VLDL was associated with the CVID specific dysbiosis index. However, there was no significant association between alpha diversity, reflecting a more general non-specific dysbiosis, and blood lipids in CVID (62). This may suggest that the association between TG and VLDL *and* gut microbiota is specific to the bacteria causing the dysbiosis in CVID. Moreover, CVID patients have increased plasma levels of LPS, a marker of gut leakage, and LPS was associated with gut dysbiosis and markers of systemic inflammation as well as T cell pathology in CVID (17, 29, 127, 128). In the study on TG and VLDL in CVID, Macpherson et al. reported a very strong correlation between LPS and TG, and LPS and VLDL (62). In the same cohort, there was no significant correlation between LPS and HDL (40), which is interesting since HDL has been shown to bind and neutralize LPS (129). However, more recent studies, suggest that LPS and related molecules are efficiently redistributed from HDL to other lipoprotein subclasses, in particular during pathological condition such as infections and inflammatory disorders (130, 131). As for TG, it is well known that bacterial endotoxins such as LPS elicits elevated plasma lipid levels due to increased synthesis and secretion of TG-rich lipoproteins by the liver and inhibition of LPL, often termed “the lipemia of sepsis” (132, 133). However, the association between TG and LPS may also reflect more indirect mechanisms. Thus, LPS is a potent and prototypical activator of TLR4, leading to increased production of inflammatory cytokines (134) that may also affect levels of TG and its major lipoprotein, VLDL. The mechanism causing the apparent shift in LPS affinity from HDL to VLDL must be further examined in more mechanistic studies and not only based on correlation analyses. Finally, and with relevance to CVID with both intestinal and hepatic pathology (15, 135), it has been suggested that intestinal synthesized HDL could be delivered into the portal circulation, thereby protecting the liver from injury in response to gut-derived LPS (24).

Another recent study showed that CVID patients had increased serum levels of bacterial DNA, belonging to gut commensals that were associated with parameters of systemic immune activation, increased serum IFN-γ and the low numbers of isotype-switched memory B cells (136). Whether these bacterial products also modulate lipid metabolisms has not been investigated. Except for one study of oropharyngeal microbial composition in CVID (137), there are to the best of our knowledge no data on dysbiosis in other organs. Forthcoming studies should address whether microbial dysbiosis outside the gut, e.g. the lungs could contribute to systemic inflammation and lipid dysregulation in CVID.

Macpherson et al. reported an association between increased plasma levels of the gut derived microbial metabolite, trimethylamine N-oxide (TMAO), *and* enhanced systemic inflammation, LPS, as well as the gut microbial abundance of *Gammaproteobacteria* in CVID patients (138). In terms of metabolic disturbances, these findings are interesting, since the TMAO-generating enzyme flavin monooxygenase 3 (FMO-3) may influence lipid metabolism and inflammation (139). Furthermore, a mice model has also suggested that TMAO may downregulate cholesterol 7α-hydroxylase, indicating a specific decline in the classical bile acid synthesis pathway with increased cholesterol levels as a consequence (140). The authors also identified the TMAO-generating enzyme FMO-3 as a powerful modifier of cholesterol metabolism and reverse cholesterol transport (140). More recently, phenylacetylglutamine has been identified as a gut microbiota-derived metabolite with impact on inflammation, thrombus formation and lipid metabolisms (141–143). In addition, SCFAs such as acetate, propionate and butyrate, mainly produced by anaerobic fermentation of gut microbes, has been associated with inflammation and lipid dysregulation in other disases (144, 145). Future studies should aim to analyze these important microbiota-derived metabolites and their relation to both metabolic and inflammatory phenotypes in CVID.

Recently, a multi-omics analysis was performed by Kaarbø et al., where duodenal biopsies with inflammation from CVID patients were compared to biopsies from healthy individuals. Pathway enrichment analyses suggested a different transcript signature for “cellular response to lipids” in CVID patients with duodenal inflammation compared to health controls (146). These findings further underscore a role for the intestine in mediating metabolic disturbances in CVID.

## Are CVID patients characterized by an increased cardiovascular risk?

**Figure 5:**
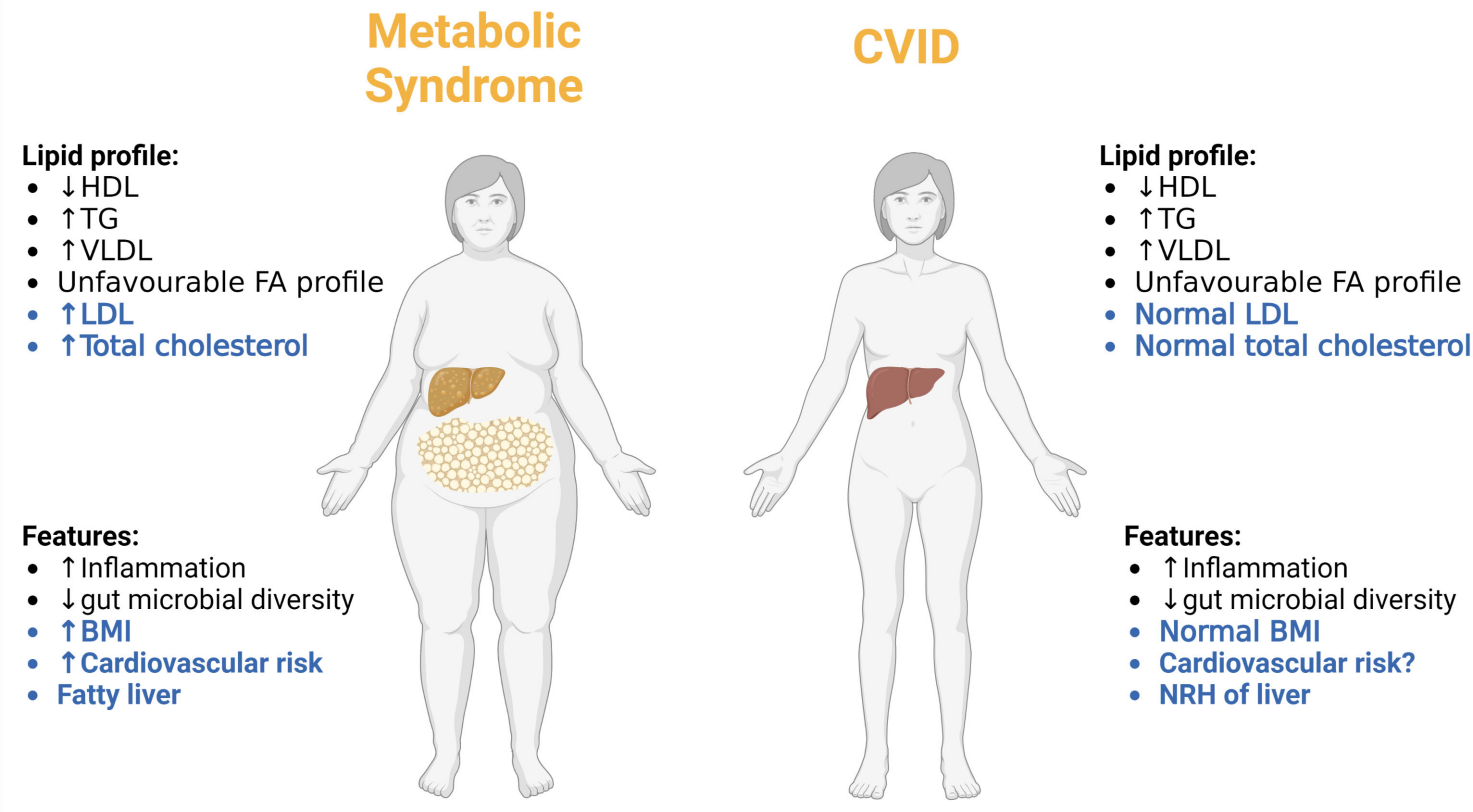
Comparing the metabolic phenotype of CVID patients to patients with classical metabolic syndrome. Differences are highlighted in blue and similarities in black. NRH, nodular regenerative hyperplasia. Created with BioRender.com.

Based on the data presented above, CVID patients are characterized by low levels of HDL and high levels of TG and VLDL as well as an unfavorable FA profile in plasma, reflecting a lipid profile which could be suggestive of increased cardiovascular risk. Indeed, it has been proposed that accelerated atherosclerosis could be overlooked in CVID (147). However, LDL levels in the CVID patients were not different from those in healthy controls, including all subclasses of this pro-atherogenic lipoprotein (62). Moreover, in the TG/VLDL study, CVID patients had similar Body Mass Index (BMI) compared to healthy controls, and within the CVID group, there was no association between TG levels *and* gender, BMI, liver pathology or diet (including energy and fat intake), all known risk factors for atherosclerotic disease (62). Also, in the FA study, there were no differences in neither the dietary intake of fat, protein, carbohydrate, meat and egg, nor the dietary intake of saturated FA, mono-unsaturated FAs, and total PUFAs, between CVID patients and the general Norwegian population, suggesting that the lipid disturbances in CVID do not merely reflect differences in diet (93). Importantly, the CVID lipid profile does not fit with the classical lipid profile in metabolic syndrome where the lipid disturbances are associated with a high BMI, or the classical high risk patients in relation atherosclerosis-related disease where high levels of LDL is an important feature (148, 149). Moreover, whereas liver steatosis is an important feature of metabolic syndrome (150), this is not the case in CVID where nodular regenerative hyperplasia is the most common cause of liver pathology, a disorder that has been suggested to involve immune mediated mechanism (151). In the TG study it was estimated that 17% of the CVID patients had a diagnosis of cardiovascular disease, which does not appear to be increased compared to the general background population (62). Of note, CVID patients with cardiovascular disease showed no difference in TG levels as compared to CVID patients without cardiovascular disease (62). Interestingly, however, whereas age is risk factor for cardiovascular diseases (152), it was the younger CVID patients (<50 years of age) that had higher TG levels than age-matched healthy controls (62). Thus, it seems that the phenotype CVID is different from those with traditional cardiovascular risk including those with metabolic syndrome (**Figure 5**). However, several studies have shown that CVID patients, and in particular the large subgroup with autoimmune and inflammatory complications, are characterized by a state of persistent low-grade non-resolving inflammation that is a well-recognized risk factor for cardiovascular disease (153). Thus, before any conclusion can be drawn in relation to the cardiovascular risk in CVID, this will have to be investigated in much larger cohorts with clinical endpoints that due to the rarity of CVID will require international multi-centre studies.

## Conclusions

**Figure 6:**
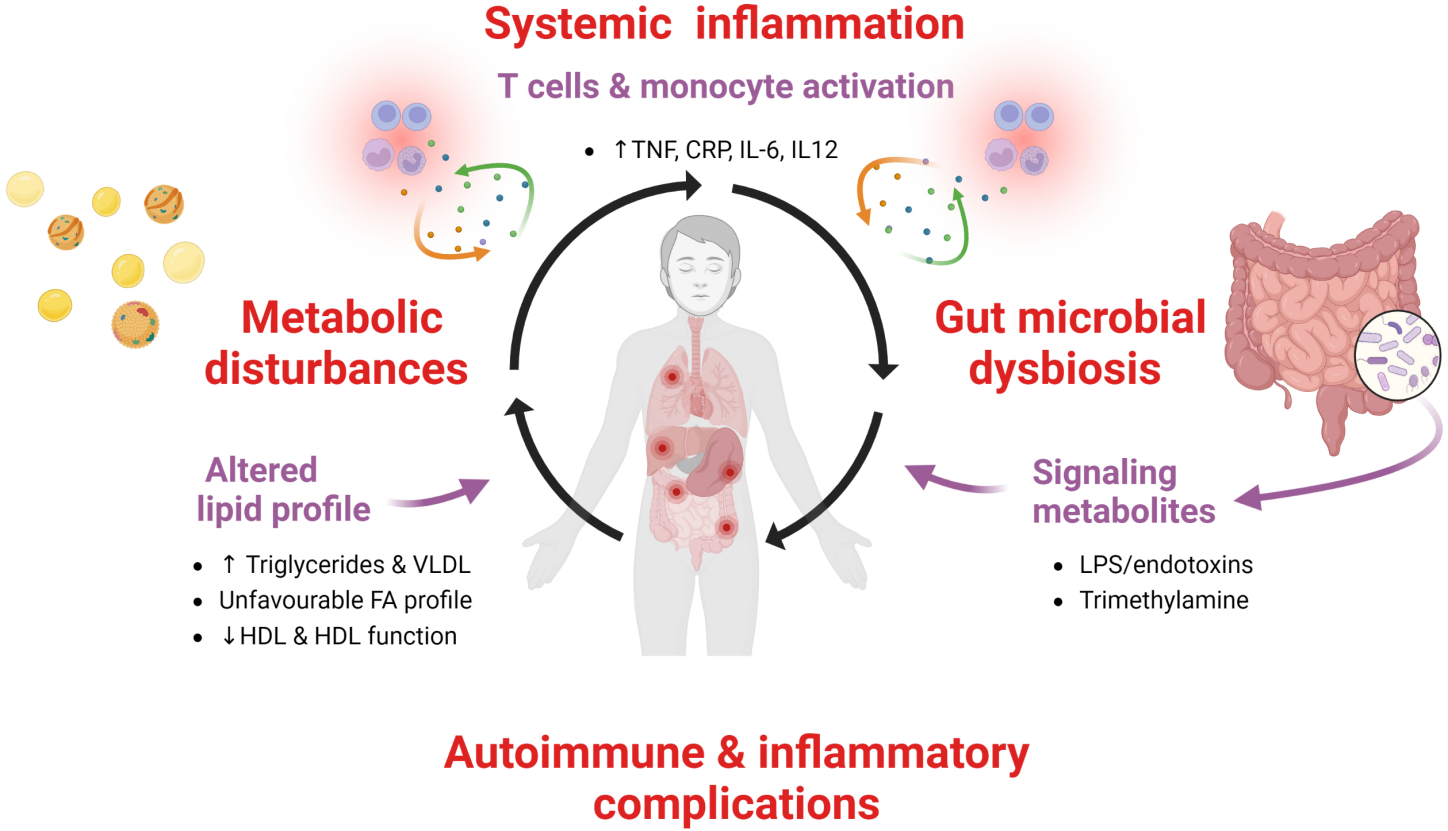
A pathogenic loop between persistent low-grade systemic inflammation, metabolic disturbances and gut microbiota in CVID. We hypothesize that in CVID patients with autoimmunity and inflammatory complications there is a pathogenic loop between persistent low-grade systemic inflammation and metabolic disturbances with altered gut microbiota as a contributing factor.

CVID patients are characterized by persistent low-grade systemic inflammation and a large proportion of them have autoimmune and inflammatory complications. Recent data, as summarized in this review article, suggest that this inflammatory phenotype is associated with a disturbed lipid profile characterized by decrease levels and impaired function of HDL, increased levels of TG and its major lipoprotein VLDL as well as an unfavourable FA profile. We hypothesize that these metabolic disturbances are linked to inflammation in a bidirectional manner with disturbed gut microbiota as a potential missing link (**Figure 6**). These interactions between metabolic and inflammatory disturbances may also reflect interaction between environmental factors and genes, i.e., epigenetic and epitranscriptomic modifications (16). However, this hypothesis needs to be confirmed by *in depth* mechanistic studies as well as studies in lager CVID populations. Further studies should also explore potential therapeutic options that could attenuate the pathogenic loop between inflammation and metabolic disturbances, such as more targeted anti-inflammatory or immune modulating therapy like inhibition of the NLRP-3 inflammasome by IL-1 inhibition, lipid modulating drugs such as ApoA-1 mimetics, cholesteryl transfer protein inhibitors, statins or diet interventions targeting the gut microbial dysbiosis in CVID.

## Author contributions

SJ and PA wrote the first draft of the paper. MM, TS, RB, BF, and BH provided intellectual input and contributed to the text. SJ made the figures in the manuscript. All authors critically revised the manuscript for important intellectual content. All authors contributed to the article and approved the submitted version.

## Glossary

**Table:**

AA	arachidonic acid
ABCA1	ATP-binding cassette transporter A1
ApoA-I	apolipoprotein A-I
ApoC-III	apolipoprotein C-III
BMI	Body Mass Index
CRP	C reactive protein
CVID	Common variable immunodeficiency
DHA	Docosahexaenoic acid
EPA	Eicosapentaenoic acid
FA(s)	Fatty acid(s)
FMO-3	flavin monooxygenase 3
HDL	high-density lipoprotein
HL	hepatic lipase
IDL	intermediate-density lipoprotein
IFN	type I interferon
IL	interleukin
LA	Linoleic acid
LDL	low-density lipoprotein
LPL	lipoprotein lipase
LPS	lipopolysaccharide
oxLDL	oxidized low-density lipoprotein
PBMC	peripheral blood mononuclear cells
PPAR	peroxisome proliferator-activated receptors
PUFAs	Polyunsaturated fatty acids
RCT	reverse cholesterol transport
ROS	reactive oxygen species, TG
SCFA	short chain FA acid
SR	scavenger receptor
TG(s)	triglyceride(s)
TLR	toll-like receptor
TMAO	trimethylamine N-oxide
TNF	Tumor necrosis factor
Treg	regulatory T cells
VLDL	very-low-density lipoprotein
XLA	X-linked agammaglobulinemia
